# Predicting the influence of multiple components on microbial inhibition using a logistic response model - a novel approach

**DOI:** 10.1186/1472-6882-14-190

**Published:** 2014-06-13

**Authors:** Cynthia J Henley-Smith, Francois E Steffens, Francien S Botha, Namrita Lall

**Affiliations:** 1 Department of Plant Science, Faculty of Natural and Agriculture Sciences, University of Pretoria, Pretoria 0002, South Africa; 2 Department of Statistics, Faculty of Natural and Agriculture Sciences, University of Pretoria, Pretoria 0002, South Africa; 3 Department of Paraclinical Sciences, Phytomedicine Programme, Faculty of Veterinary Sciences, University of Pretoria, Pretoria 0002, South Africa

**Keywords:** Synergism, Oral pathogens, Checkerboard method, *Heteropyxis natalensis*, *Melaleuca alternifolia*, *Mentha piperita*, TEAVIGO™

## Abstract

**Background:**

There are several synergistic methods available. However, there is a vast discrepancy in the interpretation of the synergistic results. Also, these synergistic methods do not assess the influence the tested components (drugs, plant and natural extracts), have upon one another, when more than two components are combined.

**Methods:**

A modified checkerboard method was used to evaluate the synergistic potential of *Heteropyxis natalensis*, *Melaleuca alternifolia*, *Mentha piperita* and the green tea extract known as TEAVIGO™. The synergistic combination was tested against the oral pathogens, *Streptococcus mutans*, *Prevotella intermedia* and *Candida albicans.* Inhibition data obtained from the checkerboard method, in the form of binary code, was used to compute a logistic response model with statistically significant results (*p* < 0.05). This information was used to construct a novel predictive inhibition model.

**Results:**

Based on the predictive inhibition model for each microorganism, the oral pathogens tested were successfully inhibited (at 100% probability) with their respective synergistic combinations. The predictive inhibition model also provided information on the influence that different components have upon one another, and on the overall probability of inhibition.

**Conclusions:**

Using the logistic response model negates the need to ‘calculate’ synergism as the results are statistically significant. In successfully determining the influence multiple components have upon one another and their effect on microbial inhibition, a novel predictive model was established. This ability to screen multiple components may have far reaching effects in ethnopharmacology, agriculture and pharmaceuticals.

## Background

Synergistic interaction between components i.e. drugs, plant and natural extracts can enhance their efficacy and bioactivity against a target. Furthermore, synergy reduces toxicity, lowers the dosage and decreases adverse side effects, as well as combating antimicrobial resistance [1,2]. Several synergistic methods and the methods used to calculate synergy, have been reviewed [3]. However, there appears to be vast discrepancies in the interpretation of synergistic results.

There is also limited information available with regards to assessing the influence multiple components have upon one another in combination. Three-component combinations have been proven successful in enhancing bioactivity [4-8]. However, the more components added in combination, the more difficult it becomes to assess the influence these components have upon each other’s bioactivity. The overall influence of the combination against the selected target would also be affected. This investigation aims to use ‘a statistical approach that would allow for a more reliable and qualitative assessment of pharmacological interactions’ [3]. The influences of multiple components upon one another and their effect on microbial inhibition were also investigated.

An indigenous South African plant, *Heteropyxis natalensis* was combined with the essential oils of *Melaleuca alternifolia* and *Mentha piperita* as well as the green tea extract known as TEAVIGO™. Combinations of these were used against the oral pathogens, *Streptococcus mutans*, *Prevotella intermedia* and *Candida albicans*[9].

## Methods

### Plant material

Aerial plant parts, comprising of leaves and twigs of *H. natalensis* was collected. The plant parts were collected from the University of Pretoria’s experimental farm during January, 2013. A voucher specimen was prepared and identified at the H.G.W.J. Schwelcherdt Herbarium (PRU), University of Pretoria. *Melaleuca alternifolia* essential oil (Holistic Emporium cc, Gauteng, South Africa), *Mentha piperita* essential oil (Holistic Emporium cc, Gauteng, South Africa), and TEAVIGO™ (Chempure (Pty) Ltd, Silverton, South Africa), were purchased for the present investigation.

### Preparation of extract

The plant material was air dried at room temperature (25°C), and ground to a fine powder using a standard food processor. The powdered material was extracted with ethanol (Merck Chemicals (Pty) Ltd Wadeville, South Africa) under pressure (100 bar) and regulated temperature of 50°C in a BUCHI Speed Extractor, E-916 (BUCHI Labortechnik AG, Switzerland). The solvent was evaporated at low boiling point in a Genevac, EZ-2 plus (Genevac SP Scientific, UK), after which the extract was subjected to antimicrobial tests.

### Microbial strains

The microorganisms used in this study included *Prevotella intermedia* (ATCC 25611), *Streptococcus mutans* (ATCC 25175) and *Candida albicans* (ATCC 10231). The bacteria were grown on Casein-peptone Soymeal-peptone (CASO) Agar (Merck Chemicals (Pty) Ltd Wadeville, South Africa) enriched with 1% sucrose (Merck Chemicals (Pty) Ltd Wadeville, South Africa) under anaerobic conditions in an anaerobic jar with Anaerocult® A (Merck Chemicals (Pty) Ltd Wadeville, South Africa), at 37°C for 48 hours. *Candida albicans* was grown on Sabouraud Dextrose 4% Agar (SDA) (Merck Chemicals (Pty) Ltd Wadeville, South Africa), at 37°C for 48 hours. Sub-culturing was done every second week. Inocula were prepared by suspending microbial test organisms in their respective broths until turbidity was compatible with McFarland Standard 1 (Merck Chemicals (Pty) Ltd Wadeville, South Africa) [10]. Therefore, the colony forming units (CFU/ml) for *P. intermedia* was 40 × 10^7^ (CFU/ml), *S. mutans* was 30 × 10^7^ (CFU/ml) and *C. albicans* was 4 × 10^7^ (CFU/ml) for each bioassay.

### Antimicrobial susceptibility testing

To determine the effects of combinations of *H. natalensis*, *M. alternifolia* essential oil, *M. piperita* essential oil and TEAVIGO™, the minimum inhibitory concentration (MIC) of each component was determined first using the anti-microbial microtiter-plate method [11]. A stock solution of the ethanol extract of *H. natalensis* was prepared in 20% dimethyl sulphoxide (DMSO) (Merck Chemicals (Pty) Ltd); while TEAVIGO™ was dissolved in distilled water. The stock solutions were serially diluted in enriched CASO broth (Merck Chemicals (Pty) Ltd) for the bacteria and Sabouraud Dextrose 4% broth (Merck Chemicals (Pty) Ltd) for *Candida*; in the 96-well microtiter-plate adding 100 μl of a McFarland Standard 1 inoculum of 48 hour old microorganisms grown at 37˚C. The final concentration of the extract and TEAVIGO™ ranged from 0.10–12.5 mg/ml and the positive control, 1.25% v/v chlorhexidine gluconate (CHX) (Dental Warehouse, Sandton, South Africa), ranged from 4.77 × 10^-6^–0.31% v/v. The essential oils were dissolved in 10% Tween (80) (Merck Chemicals (Pty) Ltd Wadeville, South Africa). The final concentration tested of the essential oils ranged from 1.6 × 10^-5^–1.25% v/v. The highest concentration of the solvents DMSO (5%) and Tween 80 (2%) were found to be non-toxic to the microorganisms tested. The inoculated plates were incubated at 37°C, under anaerobic and aerobic conditions respectively for 24 hours before adding 20 μl of the colour indicator PrestoBlue [12]. The MIC was defined as the lowest concentration that inhibited the colour change of PrestoBlue.

### Synergistic assay

The synergistic activity of the samples was determined using a modified checkerboard method. The basic design is a logarithmic design with the dosages halved at each step. Full 2-factor factorial designs were used for two factors at a time with equally spaced dosages for the other factors. The individual designs were compounded in such a way that all two-way interactions and some three-way interactions could be estimated. Two 96-well plates were prepared: the first one was used to make two-fold serial dilutions of *H. natalensis* (50 μl) in horizontal orientation, and the second plate, was used to make five-fold serial dilutions of *M. alternifolia* in the vertical orientation. Both dilutions were made in enriched CASO broth for the selected bacteria, and Sabouraud Dextrose 4% broth for the yeast, *C. albicans*. For the two-fold dilutions, 50 μl of broth was pipetted per well for the first plate and 200 μl for the five-fold dilutions in the second plate. Using a multichannel pipette, 50 μl of *M. alternifolia* was transferred to the first plate, 50 μl of the respective broth was added and then 50 μl of bacterial suspension was added to each well and incubated for 24 h at 37°C; after which 20 μl of PrestoBlue was used to indicate bacterial growth [12,13]. The concentration range of *H. natalensis* in combination ranged from 0.097 – 12.5 mg/ml, while the essential oils ranged from 1.6 × 10^-5^ – 1.25% v/v.

A third plate was prepared at the same time in the exact same manner as the first plate except that instead of 50 μl of additional broth; 50 μl of a third component, *M. piperita* was added at a sub-MIC value at a fixed concentration to all wells. The sub-MIC concentrations of *M. piperita* and TEAVIGO™ were determined on the basis of MIC values previously obtained.

This process was repeated for all the combinations of the four components for each microorganism tested.

The MIC’s of each component tested (as previously described) were also conducted at the same time acting as controls and a comparison. The concentration range of *H. natalensis* and TEAVIGO™ ranged from 0.097 – 12.5 mg/ml, while the essential oils ranged from 1.6™ 10^-5^ – 1.25% v/v. CHX was again utilized as a positive control.

Once the plates were developed with PrestoBlue, each well was assigned either a 0 to indicate no inhibition or a 1 to indicate inhibition for the logistic response model. This information was used to construct a predictive inhibition model (IBM© SPSS© version 22) for each microorganism where the antimicrobial ability of the different combinations was tested as described in the antimicrobial susceptibility testing [11].

## Results

### Antimicrobial susceptibility testing

**Figure 1:**
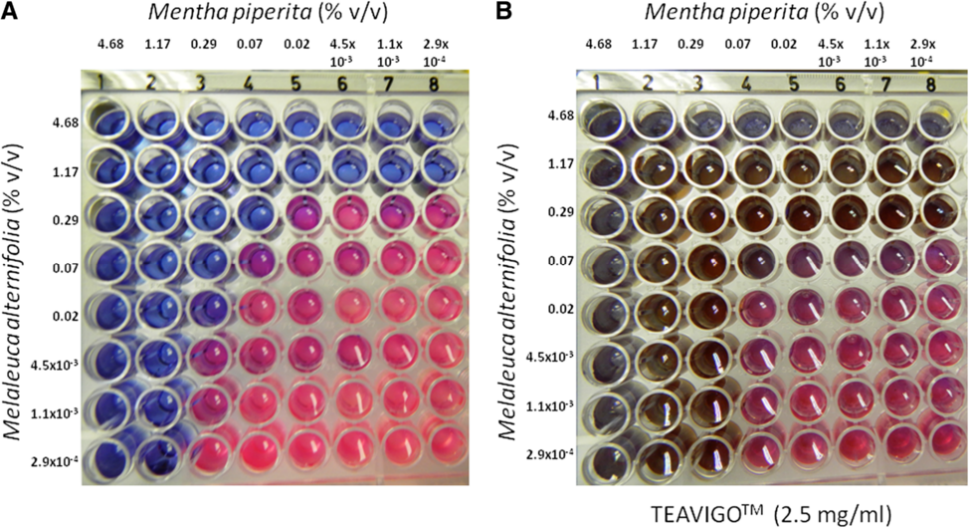
**Growth indicator, PrestoBlue, in the presence of *Prevotella intermedia*.** Plates **A** and **B** contained the essential oils *Mentha piperita* and *Melaleuca alternifolia* with Plate **B** additionally containing TEAVIGO™. Blue-green indicated inhibition of *Prevotella intermedia*, while pink-red indicated growth of *P. intermedia*.

The checkerboard method was utilized as a screening tool for the reduction of MIC values. This method also provided numerous concentration variables for the components under investigation and their inhibition potential (Figure 1). The results were converted to binary code; with 0 representing no inhibition (pink-red), and 1 representing inhibition (blue). This data was then used to compute the logistic response model.

**Figure 2:**
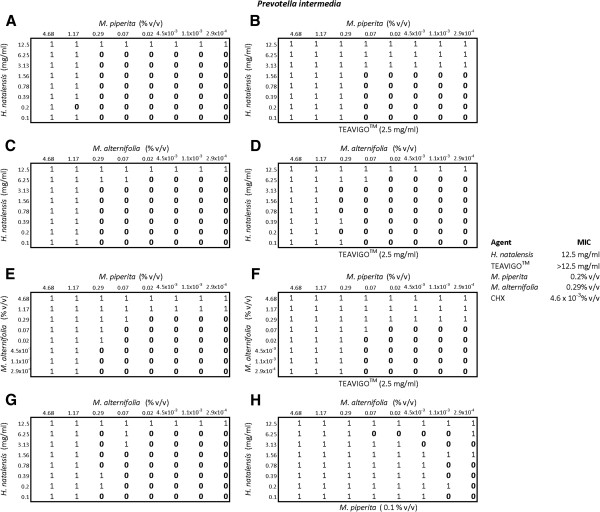
**The checkerboard results for *Prevotella intermedia*.** A 0 indicated no inhibition while 1 represented inhibition. Plates **A** and **B** contained *Mentha piperita* and *Heteropyxis natalensis*, while plate **B** had the addition of the third component, TEAVIGO™. Plates **C** and **D** contained *Melaleuca alternifolia* and *H. natalensis* with TEAVIGO™ present in plate **D**. Plates **E** and **F** contained the essential oils *M. piperita* and *M. alternifolia* with Plate **F** additionally containing TEAVIGO™. Plates **G** and **H** contained *M. alternifolia* and *H. natalensis* with the addition of *M. piperita* in plate **H**. The MIC’s for each component are also given.

In determining the antimicrobial susceptibility of *P. intermedia* (Figure 2), the addition of TEAVIGO™ (2.5 mg.ml) to plate B (of paired plates A and B) reduced the MIC of *H. natalensis* from 12.5 mg/ml to 3.13 mg/ml and that of *M. piperita* from 1.17% v/v to 0.29% v/v. In plates C and D the addition of TEAVIGO™ seemed to have little effect on either *H. natalensis* or *M. alternifolia*; however, when TEAVIGO™ was added to the essential oils, *M. piperita* and *M. alternifolia* (plates E and F) both essential oils MIC’s were reduced from 1.17% v/v to 0.29% v/v overall. In plates G and H with *H. natalensis* and *M. alternifolia* and the addition of *M. piperita* in plate H there was a significant decrease in both components MIC’s. Even though the pattern of inhibition to no-inhibition was a little scattered, the overall reduction of the MIC of *H. natalensis* from 12.5 mg/ml to 1.56 mg/ml and for *M. alternifolia* from 1.17% v/v to 4.5 × 10^-3^% v/v was obtained. There is a significant increase in the antimicrobial activity of the components in combination when compared to the MIC values of the individual components.

**Figure 3:**
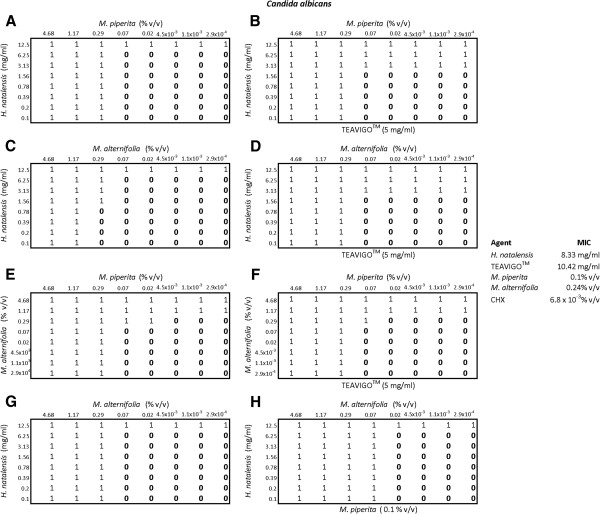
**The checkerboard results for *Candida albicans.*** A 0 indicated no inhibition while 1 represented inhibition. Plates **A** and **B** contained *Mentha piperita* and *Heteropyxis natalensis*, with plate **B** having the addition of the third component, TEAVIGO™ (5 mg/ml). Plates **C** and **D** contained *Melaleuca alternifolia* and *H. natalensis* with TEAVIGO™ present in plate **D**. Plates **E** and **F** contained the essential oils *M. piperita* and *M. alternifolia* with Plate **F** additionally containing TEAVIGO™. Plates **G** and **H** contained *M. alternifolia* and *H. natalensis* with the addition of *M. piperita* in plate **H**. The MIC’s for each component are also given.

With *C. albicans* (Figure 3), the addition of TEAVIGO™ at a sub-MIC (5 mg/ml) in plate B, reduced the MIC of *H. natalensis* from 12.5 mg/ml to 3.13 mg/ml but had no impact on the MIC of *M. piperita*. The MIC of *H. natalensis* was again reduced in plate D and the essential oil *M. alternifolia* was also reduced from 1.17% v/v to 0.29% v/v. There was virtually no difference in plates E and F containing *M. alternifolia* and *M. piperita* with the addition of TEAVIGO™. The addition of *M. piperita* in plate H reduced the MIC of *M. alternifolia* from 0.29% v/v to 0.07% v/v but had no effect on the MIC of *H. natalensis*.

*Heteropyxis natalensis* on its own inhibited *C. albicans* at 8.33 mg/ml; in combination with *M. piperita* and TEAVIGO™ this concentration was reduced to 3.13 mg/ml. *Melaleuca alternifolia* in combination with *H. natalensis* and *M. piperita* reduced the MIC from 0.24% v/v (of *M. alternifolia* on its own) to 0.07% v/v.

**Figure 4:**
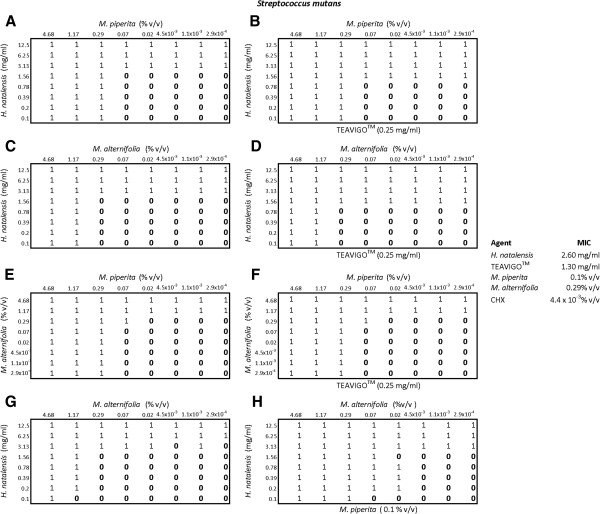
**The checkerboard results for *Streptococcus mutans*.** A 0 indicated no inhibition while 1 represented inhibition. Plates **A** and **B** contained *Mentha piperita* and *Heteropyxis natalensis* were paired, with plate **B** having the addition of the third component, TEAVIGO™. Plates **C** and **D** contained *Melaleuca alternifolia* and *H. natalensis* with TEAVIGO™ present in plate **D**. Plates **E** and **F** contained the essential oils *M. piperita* and *M. alternifolia* with Plate **F** additionally containing TEAVIGO™. Plates **G** and **H** contained *M. alternifolia* and *H. natalensis* with the addition of *M. piperita* in plate **H**. The MIC’s for each component are also given.

In determining the inhibitory effect of three components on *S. mutans* (Figure 4), there was a reduction in the MIC of *H. natalensis* from 3.13 mg/ml to 1.56 mg/ml with the addition of TEAVIGO™ but there was no effect on *M. piperita* (plates A and B). The same effect was exhibited with *H. natalensis*, *M. alternifolia* and the addition of TEAVIGO™ in plates C and D. There was no apparent effect of TEAVIGO™ on the essential oils, *M. piperita* and *M. alternifolia* (plates E and F). There was a marked increase in the inhibition by *M. alternifolia* with the addition of *M. piperita*; from 1.17% v/v to 0.02% v/v (plates G and H). *Melaleuca alternifolia* on its own inhibited *S. mutans* at 0.29%. In combination with *H. natalensis* and *M. piperita* this inhibitory concentration was reduced to 0.02% v/v.

### Logistic response model

The logistic response model [14] was used to predict the probability associated with each value of the binary response (Tables 1, 2, 3, 4, 5, 6, 7, 8 and 9). A stepwise procedure [15] was used to select the most important predictors. In this investigation the probability of inhibition was modeled.

**Table 1:** **Logistic model summary for** ***Prevotella intermedia***

**-2 Log likelihood**	**Nagelkerke R square**^**a**^
215.765	.864

^a^The Nagelkerke R Square is the logistic regression equivalent of the usual coefficient of determination used in multiple linear regression [16].

**Table 2:** **Classification table for *Prevotella intermedia***

**Observed**		**Predicted**		
		**Y**		**Percentage correct**
		**0**	**1**	
Y	0	267	17	94.0
	1	20	336	94.4
Overall Percentage				94.2

**Table 3:** **Variables in the equation for** ***Prevotella intermedia***

	**B**^**a**^	**S.E.**^**b**^	**Sig.**^**c**^
X1	.662	.072	.000
X2	82.473	11.501	.000
X3	60.709	7.536	.000
X4	1.068	.220	.000
X1 by X3	-4.795	.690	.000
X3 by X4	35.518	18.520	.055
Constant	-6.100	.638	.000

^a^Regression Co-efficient.

^b^Standard Error.

^c^Significance.

**Table 4:** **Logistic model summary for *Candida albicans***

**-2 Log likelihood**	**Nagelkerke R square**
92.432	.947

**Table 5:** **Classification table for *Candida albicans***

**Observed**		**Predicted**		
		**Y**		**Percentage correct**
		**0**	**1**	
Y	0	268	9	96.8
	1	13	350	96.4
Overall percentage				96.6

**Table 6:** **Variables in the equation for *Candida albicans***

	**B**^**a**^	**S.E.**^**b**^	**Sig.**^**c**^
X1	3.488	.852	.000
X2	770.772	207.145	.000
X3	773.135	212.726	.000
X4	5.946	1.620	.000
X1 by X3	-61.807	17.021	.000
X2 by X4	-116.903	32.381	.000
X3 by X4	-4.824	2.252	.032
Constant	-39.892	10.602	.000

^a^Regression Co-efficient.

^b^Standard Error.

^c^Significance.

**Table 7:** **Logistic model summary for *Streptococcus mutans***

**-2 Log likelihood**	**Nagelkerke R square**
162.446	.900

**Table 8:** **Classification table for *Streptococcus mutans***

**Observed**		**Predicted**		
		**Y**		**Percentage correct**
		**0**	**1**	
Y	0	251	13	95.1
	1	23	353	93.9
Overall percentage				94.4

**Table 9:** **Variables in the equation for *Streptococcus mutans***

	**B**^**a**^	**S.E.**^**b**^	**Sig.**^**c**^
X1	2.249	.253	.000
X2	34.504	5.160	.000
X3	55.328	7.499	.000
X4	12.302	2.154	.000
X1 by X3	-5.628	.791	.000
Constant	-6.086	.666	.000

^a^Regression Co-efficient.

^b^Standard Error.

^c^Significance.

The response variable is Y as follows:

Y = 0 means no response

Y = 1 means inhibition

*X* = (X_1_,X_2_,X_3_,X_4_) is the combination of dosages with X_1_ representing *H. natalensis*, X_2_ - *M. alternifolia*, X_3_ - *M. piperita* and X_4_ - TEAVIGO™

p(*X*) = the probability of inhibition given the dosage combination

O(*X*) is the odds of obtaining inhibition

$$O\left(X\right)=\frac{p\left(X\right)}{1-p\left(X\right)}$$

The log(odds) is LN{O(*X*)}

The logistic regression model is a linear model (linear in terms of the regression coefficients) that links the log (odds) to the dosages and to interaction terms between the dosages.

The function is estimated by means of maximum likelihood. In this case (Table 3), the estimate is

$$\begin{array}{l} \;LN\left\{O,(,X,)\right\}=-6.1+0.662X_{1}+82.473X_{2}+60.709X_{3}+\\ 1.068X_{4}-4.795X_{1}X_{3}+35.518X_{3}X_{4} \end{array}$$

The estimated odds of inhibition is then

$$O\left(X\right)=\mathrm{EXP}\left(\mathrm{LN}\left(O\left(X\right)\right)\right)=e^{\mathrm{LN}\left(O\left(X\right)\right)}$$

The estimated probability of inhibition is then

$$p\left(X\right)=\frac{O\left(X\right)}{1+O\left(X\right)}$$

### Validation of the models

With the variables in the equation for *Prevotella intermedia* (Table 3), 80% of the original sample was randomly selected to be the training sample, and the remaining 20% formed the test sample. The model was fitted using the training sample and used to predict the outcomes of the training and validation samples. The outcome was that 92.4% of the training sample was correctly classified and 95.4% of the validation sample was correctly classified. This is considered satisfactory.

With the variables in the equation for *Candida albicans* (Table 6), 80% of the original sample was randomly selected to be the training sample, and the remaining 20% formed the test sample. The model was fitted using the training sample and used to predict the outcomes of the training and validation samples. The outcome was that 96.8% of the training sample was correctly classified and 95.7% of the validation sample was correctly classified. This is considered satisfactory.

With the variables in the equation for *Streptococcus mutans* (Table 9), 80% of the original sample was randomly selected to be the training sample, and the remaining 20% formed the test sample. The model was fitted using the training sample and used to predict the outcomes of the training and validation samples. The outcome was that 92.7% of the training sample was correctly classified and 96.7% of the validation sample was correctly classified. This is considered satisfactory.

### Predictive inhibition model

A maximum of three components were tested on a microtitre plate using the modified checkerboard method. All possible combinations of the four components were tested this way (Figure 2). The log odds estimate, LN{O(*X*)}, obtained from the logistic regression model, combines the data of the four components in the predictive inhibition model. This enabled the probability of inhibition to be calculated utilizing all four components. The predictive inhibition model also provided information on the influence, the different components tested, had upon one another and on the probability of inhibition (Table 10).

**Table 10:** **The influence each component had on the probability of inhibition in the predictive model for *Prevotella intermedia***

***H*. *natalensis *** **(mg/ml)**	***M*. *alternifolia *** **(% v/v)**	***M*. *piperita *** **(% v/v)**	**TEAVIGO™ (mg/ml)**	**p**
3.125				0.017442
3.125	0.05			0.523084
3.125	0.05	0.05		0.915183
3.125	0.05	0.05	5	1.000000

Tables 10, 11 and 12, show further validation of the predictive models for each microorganism tested. The models were used to predict the probability of inhibition outside the experimental area, and additional experiments were performed in the laboratory to judge the performance of the models. The performance was satisfactory.

**Table 11:** **Predictive model showing the probability of inhibition (p) for *Prevotella intermedia***

***H*. *natalensis *** **(mg/ml)**	***M*. *alternifolia *** **(% v/v)**	***M*. *piperita *** **(% v/v)**	**TEAVIGO™ (mg/ml)**	**p**	**Outcome**
0.78125	0.002	0.002	1.25	0.02023	No inhibition
1.5625	0.002	0.01	1.25	0.06981	No inhibition
1.5625	0.01	0.01	1	0.09233	No inhibition
1.5625	0.01	0.01	2.5	0.46238	No inhibition
3.125	0.01	0.05	2.5	0.99795	No inhibition
3.125	0.05	0.05	2	0.99969	Inhibition
3.125	0.05	0.05	5	1.00000	Inhibition
6.25	0.05	0.25	5	1.00000	Inhibition
6.25	0.25	0.25	4	1.00000	Inhibition

**Table 12:** **Predictive model showing the probability of inhibition (p) for *Candida albicans***

***H*. *natalensis *** **(mg/ml)**	***M*. *alternifolia *** **(% v/v)**	***M*. *piperita *** **(% v/v)**	**TEAVIGO™ (mg/ml)**	**p**	**Outcome**
0.78125	0.002	0.002	2.5	0.00000	No inhibition
1.5625	0.002	0.01	2	0.00000	No inhibition
1.5625	0.01	0.01	2	0.00003	No inhibition
1.5625	0.01	0.01	5	0.97528	No inhibition
3.125	0.01	0.05	4	1.00000	Inhibition
3.125	0.05	0.05	4	1.00000	Inhibition
3.125	0.05	0.05	10	1.00000	Inhibition
6.25	0.05	0.25	8	1.00000	Inhibition
6.25	0.25	0.25	8	1.00000	Inhibition

Based on the predictive inhibition model where 1 indicates the probability for 100% inhibition; *P. intermedia* (Table 11), *C. albicans* (Table 12) and *S. mutans* (Table 13) were successfully inhibited. At probabilities lower than 100% almost no inhibition was obtained for *P. intermedia* and *C. albicans*, while there was inhibition of *S. mutans* at 99%. *Prevotella intermedia* seemed to be sensitive to the concentration of *M. alternifolia*, as no inhibition was obtained when *M. alternifolia* was decreased to 0.01% v/v (at a 99.8% probability).

**Table 13:** **Predictive model showing the probability of inhibition (p) for *Streptococcus mutans***

***H*. *natalensis *** **(mg/ml)**	***M*. *alternifolia *** **(% v/v)**	***M*. *piperita *** **(% v/v)**	**TEAVIGO™ (mg/ml)**	**p**	**Outcome**
0.390625	0.00008	0.0004	0.390625	0.40661	No inhibition
0.390625	0.0004	0.0004	0.390625	0.40928	No inhibition
0.390625	0.002	0.002	0.390625	0.44355	No inhibition
0.78125	0.0004	0.002	0.78125	0.99549	Inhibition
0.78125	0.002	0.002	0.78125	0.99573	Inhibition
0.78125	0.01	0.01	0.78125	0.99784	Inhibition
1.5625	0.002	0.01	1.5625	1.00000	Inhibition
1.5625	0.01	0.01	1.5625	1.00000	Inhibition
1.5625	0.05	0.05	1.5625	1.00000	Inhibition

There is a reduction in the MIC values of the individual components, when used in combination for each of the microorganisms tested (Table 14). And therefore, we can state that there is an overall increase in the inhibitory activity when the components are used in combination.

**Table 14:** Comparison of the minimum inhibitory concentrations of the tested components, individually and in combination, after utilizing the predictive model

	***P*. *intermedia***		***C*. *albicans***		***S*. *mutans***	
	**Alone**^**a**^	**Combo**^**b**^	**Alone**^**a**^	**Combo**^**b**^	**Alone**^**a**^	**Combo**^**b**^
***H*. *natalensis *** **(mg/ml)**	12.50	3.13	8.33	3.13	2.60	0.78
**TEAVIGO™ (mg/ml)**	>12.5	2.00	10.42	4.00	1.30	0.78
***M*. *piperita *** **(% v/v)**	0.20	0.05	0.10	0.05	0.10	2 × 10^-3^
***M*. *alternifolia *** **(% v/v)**	0.29	0.05	0.24	0.01	0.29	4 × 10^-4^

^a^Component tested individually.

^b^Components tested in combination.

## Discussion

The synergistic combination of the components had different effects on each of the microorganisms tested. This may indicate the possible mechanism of action of these components. The combinations of *M. piperita*, *H. natalensis* and TEAVIGO™, against *P. intermedia*, *C. albicans* and *S. mutans* all had similar outcomes, resulting in an increased *H. natalensis* activity against these microorganisms (plates A and B of Figures 2, 3 and 4). The combination of *M. alternifolia*, *H. natalensis* and TEAVIGO™ (plates C and D) resulted in an increase in the activity of *H. natalensis* against *S. mutans* and both *H. natalensis* and *M. alternifolia* against *C. albicans*. However, this combination had no apparent effect on *P. intermedia*. The reverse situation occurred for the combination of *M. piperita*, *M. alternifolia* and TEAVIGO™ (plates E and F), where an increase in antimicrobial activity was noted for *M. piperita* and *M. alternifolia* on *P. intermedia* but there were no noticeable effects on *C. albicans* and *S. mutans*. Both Gram-positive and Gram-negative bacteria's cell walls consist of peptidoglycan. Peptidoglycan is comprised of *N*-acytyl-muramic acid and *N*-acetyl-glucosamine cross linked by peptide side chains and cross-bridges; however, peptidoglycan is thicker in Gram-positive bacteria. Gram-negative bacteria also possess a periplasmic space which lies between the outer membrane and the cytoplamic membrane. It is within this space that some Gram-negative bacteria produce the lactamase enzyme that can resist drugs such as penicillin [17]. The combination of *M. piperita*, *M. alternifolia* and TEAVIGO™ may target the transenvelope efflux pump in *P. intermedia* which does not occur in *S. mutans* or the eukaryotic *C. albicans*[18]. The combination of *M. piperita*, *M. alternifolia* and *H. natalensis* (plates G and H) all resulted in an increase in *M. alternifolia* antimicrobial activity but only on *P. intermedia* was the activity of *H. natalensis* also increased. Overall it would seem that TEAVIGO™ increases the antimicrobial inhibitory activity of *H. natalensis*; while *M. piperita* has a similar effect on its essential oil counterpart *M. alternifolia*.

The predictive inhibition model provides information of the influence the different components tested have upon one another and on the probability of inhibition. This ‘determination of influence’ goes beyond the classification of synergism, indifference and antagonism. A probability of inhibition value was assigned to the concentration of each individual component and in various combinations of two to four. The concentrations of each component can then be adjusted to obtain a 100% probability of inhibition. The predictive inhibition model is also based on statistically significant results (*p* < 0.05) from the logistic response model. This has reduced the need to calculate the fractional inhibitory concentration (FIC) or equivalent values.

There is a reduction in the MIC values of each individual component, when used in combination for each of the microorganisms tested (Table 14). Therefore, we can state that there is an overall increase in the inhibitory activity when the components are used in combination.

## Conclusions

The use of the checkerboard method as a screening tool, utilizing the binary code to indicate inhibition and no inhibition and the input of those results into a logistic response model, lead to the successful construction of a predictive inhibition model. The predictive model not only gives the probability of 100% inhibition; but also shows the influence of those components upon one another and their ability to inhibit microbial growth.

The applications of this technique are almost limitless. Not only can the inhibitory effect of different plants in combinations of more than two be determined; new multiple drug combinations can be screened too. In ethnopharmacology, where the remedies of traditional healers are tested this technique will also be useful as the healers often use combinations of a variety of different plants for a treatment. In Agriculture new pesticides can also be screened as the combination of multiple components leads to the slower development of resistance.

## Abbreviations

ATCC: American type culture collection; CASO: Casein-peptone Soymeal-peptone; CFU: Colony forming units; CHX: Chlorhexidine gluconate; DMSO: Dimethyl sulphoxide; FIC: Fractional inhibitory concentration; MIC: Minimum inhibitory concentration; PRU: H.G.W.J. Schwelcherdt Herbarium; v/v: Volume per volume.

## Competing interests

The University of Pretoria holds a provisional South African patent (ZA2013/06534) relating to the content of the manuscript. No financial benefits have been received by the authors.

## Authors’ contributions

CJHS conceived the study, carried out the experimentation, and drafted the manuscript. FES conducted the statistical analysis and edited the manuscript. FSB and NL supervised the project and edited the manuscript. All the authors read and approved the final manuscript.

## Pre-publication history

The pre-publication history for this paper can be accessed here:

http://www.biomedcentral.com/1472-6882/14/190/prepub
